# Injectables and Facelifts: Can We Coexist? A Retrospective Chart Review Assessing Injectable Treatments Preceding and Following Rhytidectomy

**DOI:** 10.1111/jocd.70690

**Published:** 2026-02-09

**Authors:** Bridget Myers, Maya Firsowicz, Payvand Kamrani, Steven Dayan, Sabrina Fabi

**Affiliations:** ^1^ Department of Dermatology UC Davis Sacramento California USA; ^2^ Cosmetic Laser Dermatology San Diego California USA; ^3^ Department of Otolaryngology University of Illinois Chicago Illinois USA

**Keywords:** biostimulators, complications, energy‐based devices, facelift, filler, injectables, rhytidectomy

## Abstract

**Background:**

Facelift surgery is a cornerstone of facial rejuvenation, yet it may not fully address volume loss, skin quality, or other age‐related changes. Minimally invasive procedures—such as injectables and energy‐based devices—are increasingly utilized before and after facelift surgery, though their impact on surgical outcomes remains uncertain.

**Objective:**

The objective of this single‐center retrospective pilot study is to describe patterns of minimally invasive procedures among patients undergoing facelift surgery in a real‐world clinical setting and identify areas for future investigations regarding their potential influence on surgical outcomes.

**Methods:**

We analyzed data from 20 patients who underwent facelift surgery and had a documented history of neurotoxin, filler, biostimulator, or energy‐based device treatment. Data included patient demographics, treatment history, surgical details, and post‐operative satisfaction. Given the limited sample size and retrospective design, the analyses done were descriptive and exploratory in nature.

**Results:**

All patients received neurotoxin prior to facelift, 90% received hyaluronic‐acid filler, and 55% received poly‐L‐lactic acid or calcium hydroxylapatite. Following surgery, all patients resumed neurotoxin use, and 60% received filler treatments within the first post‐operative year. No reported significant surgical complications occurred because of prior filler or biostimulator treatments, and patient satisfaction was high for facelift outcomes.

**Conclusion:**

Within our small, retrospective cohort, prior injectable or energy‐based device use was not associated with an increased risk of observable adverse surgical outcomes; however, the limited sample size precludes definitive assessment of safety or risk. Larger, prospectively designed studies are warranted to validate these early observations and optimally define best practices for combining surgical and nonsurgical rejuvenation.

## 1 Introduction

Facial rejuvenation is one of the most sought‐after aesthetic goals. As we age, a dynamic combination of physiologic changes occurs, which include a reduction in facial fat volume, structural skeletal support, and skin elasticity and thickness. These changes result in a gradual descent of soft tissues contributing to sagging, jowling, and contour irregulatories [1].

Facelift surgery remains a cornerstone for correcting facial laxity. However, surgery alone often cannot fully address the changes in skin quality, texture, and volume depletion, which accompany aging. To complement these limitations, nonsurgical modalities—namely, injectables (neurotoxins, fillers, and biostimulators), lasers, and energy‐based devices (EBDs)—have become integral components of modern aesthetic practice. Most contemporary patients now pursue facial rejuvenation through a personalized combination of surgical and nonsurgical treatments tailored to their individual goals and evolving needs over time.

For example, in a review of 157 patients who underwent a facelift before the age of 50, 32% had received injectables prior to surgery, 16% laser resurfacing, and 10% EBDs [2]. On average, patients felt they looked 4–5 years younger after their nonsurgical treatments and approximately 8 years younger after their facelift. Notably, most did not regret the money spent on less invasive procedures before facelift surgery, highlighting the growing public interest in multimodal approaches to facial rejuvenation [2].

As surgical and nonsurgical techniques increasingly overlap in clinical practice, considerable discussion has arisen regarding how prior injectable or device treatments may influence surgical planning and facelift outcomes. A survey of Aesthetic Society members revealed that 51.9% of respondents perceived a history of pan‐facial filler injections as increasing the technical complexity of facelift surgery [3]. This was most commonly attributed to scarring or fibrosis distorting tissue planes or hindering healthy flap elevation. Additionally, 39.7% of surgeons reported observing higher rates of post‐operative complications in patients with a history of pan‐facial filler injections. Commonly cited issues included palpable or visible filler material (32.7%), compromised flap vascularity (15.4%), and a perceived decrease in the overall longevity of the facelifting effect (9.6%) [3].

Despite these observations, limited objective data exist regarding the outcomes of facelifts in patients with a history of facial fillers and EBD treatments. The demand for these therapies after a facelift is similarly unknown. The present retrospective pilot study was designed to characterize real‐world patterns of injectable and EBD use before and after facelift surgery and explore preliminary trends in post‐operative outcomes. By describing these patterns, our goal is to generate early insights that may inform future, larger investigations aimed at developing practice parameters for integrating surgical and nonsurgical facial rejuvenation.

## 2 Methods

This is a single center retrospective pilot study of 20 patients from a busy cosmetic practice who underwent facelift surgery after establishing care at our clinic. Data were collected between December 2024 and June 2025. Only those who had already established care at our practice and subsequently underwent facelift surgery were eligible for inclusion.

All eligible patients provided the approximate date of their facelift and their age at that time. They completed a brief survey regarding their surgical experience, including satisfaction, downtime, and any related complications (Appendix S1). Whether fat grafting was performed concurrently and whether filler was dissolved prior to facelift was recorded (Table 1). Menopausal status and the use of hormone replacement therapy at the time of the facelift surgery were also included.

**TABLE 1:** Patient demographics and survey responses (*n* = 20).

Female gender	20 (100%)
Average age at time of facelift	55.95 years‐of‐age
Menopausal at time of facelift	14 (78%, 18 responded)
Taking hormone replacement at time of facelift	6 (43%, 18 responded)
Aesthetic goal achieved with facelift	16 (80%)
Would receive facelift again	16 (80%)
Fat transfer done at time of facelift	9 (45%)
Filler not dissolved prior to procedure	16 (100%, 16 responded)

Electronic medical records were reviewed to collect data on minimally invasive procedures, including neurotoxins, fillers, biostimulators, and EBDs, performed before and/or after facelift surgery (Table 2). Per patient, we documented the duration of injectable use prior to surgery, total number of injectable sessions, and estimated time in years between their first filler or biostimulator treatment and their facelift (Table 3). We also calculated the number of days between the date of their facelift and the date of subsequent neurotoxin, filler, biostimulator, and/or EBD treatment, if applicable (Table 4). The location treated and the product used were also recorded.

**TABLE 2:** Aesthetic treatments received before and after facelift surgery.

Treatment type	Received before facelift	Received after facelift
Neuromodulator	20 (100%)	20 (100%)
Filler	18 (90%)	12 (60%)
PLLA	11 (55%)	7 (35%)
MFUV	13 (65%)	3 (15%)

Abbreviations: MFUV = microfocused ultrasound visualization; MRF = monopolar radiofrequency; PLLA = poly‐L‐lactic acid.

**TABLE 3:** Average number of years our patient cohort received injectables prior to facelift.

	Average time (years)
Duration of injectable treatments prior to facelift (total)	7.55
Duration of HA filler treatments prior to facelift	7.11
Duration of biostimulator treatments prior to facelift	5.42

**TABLE 4:** Time elapsed between last aesthetic treatment and facelift.

	Median time (days)	Minimum (days)	Maximum (days)
Time elapsed between last neuromodulator and facelift	202.5	20	1078
Time elapsed between last HA filler and facelift	272.5	93	1348
Time elapsed between last biostimulator and facelift	561	171	2147
Time elapsed between last microfocused ultrasound treatment and facelift	380	244	2262

Given the small sample size and retrospective design, analyses done were descriptive and exploratory in nature. Descriptive statistics (mean, median, and frequency) were used to summarize trends in injectables and EBDs within our cohort of patients with a history of facelift surgery.

## 3 Results

The average patient in our study had been receiving injectable treatments for 7.55 years prior to facelift surgery, with 100% receiving neuromodulator injections, 90% receiving hyaluronic acid (HA) fillers, and 55% receiving biostimulatory fillers. The duration of HA filler treatments prior to facelift ranged from 2 to 17 years, with an average of 7 years of consistent HA injectables prior to facelift surgery (Table 3). The duration of poly‐L‐lactic acid (PLLA) treatments prior to facelift surgery ranged from 1 to 9, with an average of 5.42 years. Patients received on average 6.35 sessions of HA filler and 3.15 sessions of PLLA prior to their facelift. 65% of our patients had received micro‐focused ultrasound (MFU) prior to their facelift, averaging 2.38 treatment sessions. Patients received their most recent neuromodulator injection a median of 202.5 days prior to facelift surgery (Table 4). They received HA filler a median of 272.5 days prior to facelift surgery; PLLA 561 days prior; and MFU‐V 380 days prior to facelift. The majority of patients (78%) within our cohort were post‐menopausal at the time of facelift (*n* = 14 out of 18 surveyed). When surveyed about hormone replacement therapy, 6 out of 14 were on hormone replacement at the time of facelift, whereas 8 out of 14 were not.

Within our patient cohort, multiple surgeons performed the patients' facelift, and the majority were deep plane facelifts. Four out of 20 patients had previously undergone facelift prior to establishing care with us, undergoing a second facelift during their time with our practice. For these patients, data were collected in reference to their second facelift. Overall, patients were very satisfied with their facelift, with an average satisfaction score of 4.25 (scale 1–5). 80% of patients reported achieving their aesthetic goals and indicated that they would undergo the facelift again. 45% of patients received a fat transfer during their facelift. Reported complications included scarring (two patients, 10%), prolonged swelling (one patient), prolonged bruising (one patient), and infection (one patient). Both patients who reported scarring had undergone multiple facelift surgeries. Average downtime following facelift was approximately 16.8 days. Of the 16 patients queried regarding filler dissolution, none reported requiring filler removal prior to facelift.

Following facelift, all patients (100%) presented for neuromodulator treatment with a median interval of 177 days (Table 5). Additionally, 60% of patients presented for filler and 35% for biostimulator injections, with median intervals of 300 days and 492 days, respectively. One patient was excluded from timeline calculations because of a delay in follow‐up during the COVID‐19 pandemic.

**TABLE 5:** Time elapsed between facelift and subsequent injectable treatment.

	Median time (days)	Minimum (days)	Maximum (days)
Time between facelift and subsequent neuromodulator treatment	177	31	1370
Time between facelift and subsequent filler treatment	300	31	979
Time between facelift and subsequent biostimulator treatment	492	31	2155

**FIGURE 1:**
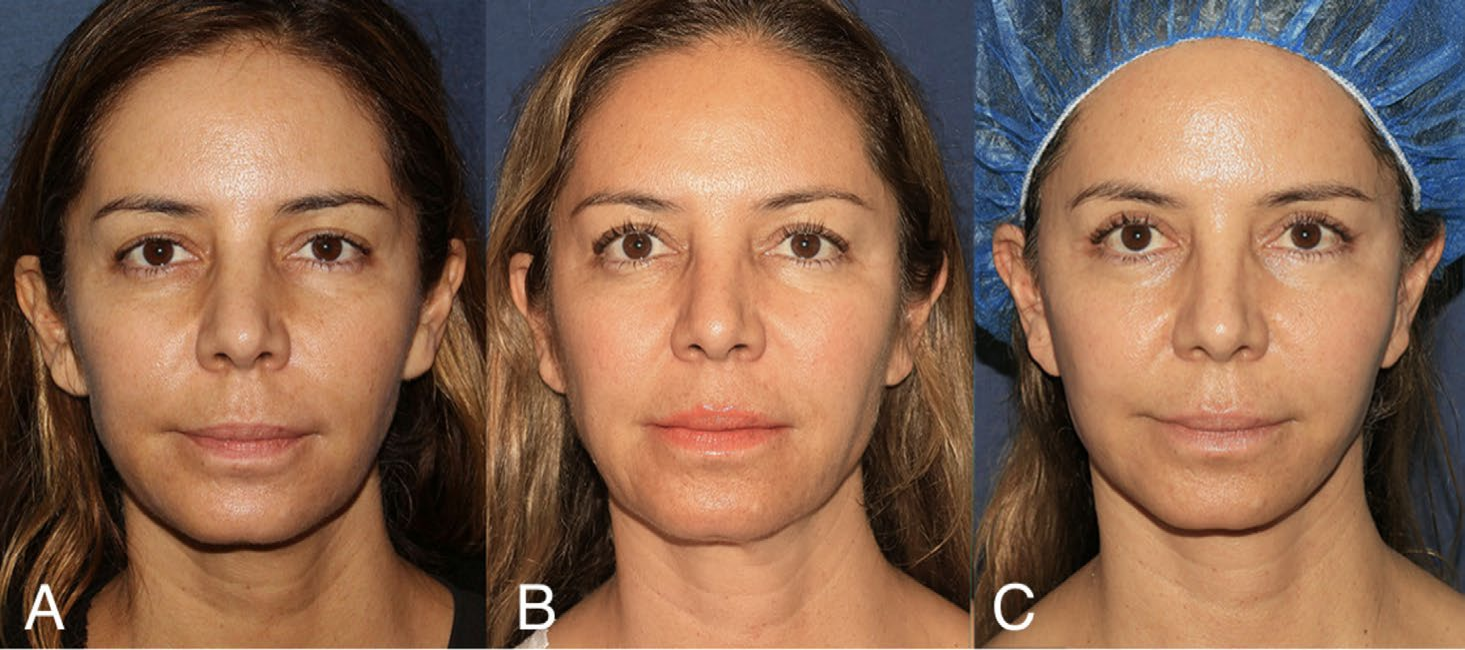
Left to right: (A) 46‐year‐old at patient's initial consultation. (B) Patient, now 53‐years‐old, after 7 years of receiving regular injectable and MFU‐V treatments (prior to facelift surgery). (C) Patient, now 54‐years‐old, 3 months after receiving facelift surgery.

**FIGURE 2:**
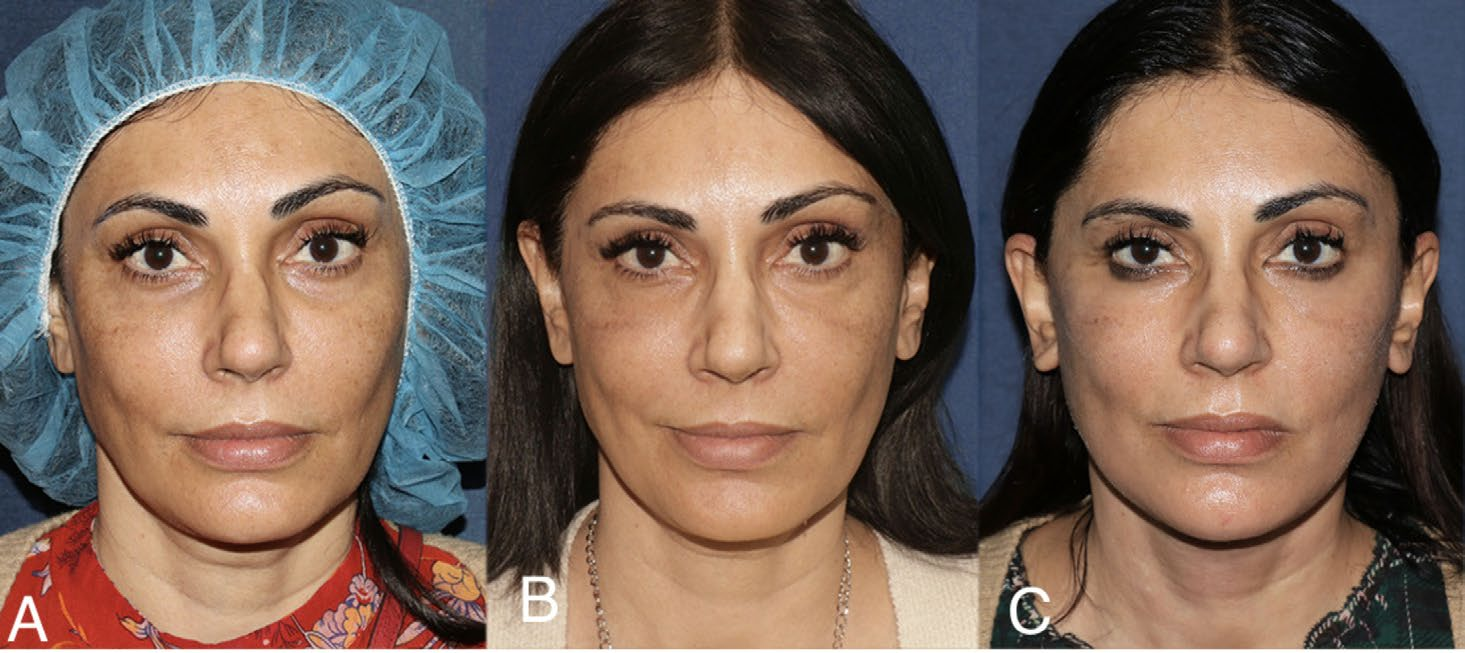
Left to right: (A) 48‐year‐old at patient's initial consultation. (B) Patient, now 50‐years‐old, after 2 years of receiving regular injectables and MFU treatments (prior to facelift surgery). (C) Patient, now 51‐years‐old, 2 months after undergoing facelift surgery.

Representative clinical photographs are shown in Figures 1 and 2, demonstrating patients' appearance at baseline, after consistent injectable and MFU treatments, and following facelift surgery.

## 4 Discussion

Among all nonsurgical procedures in aesthetic dermatology, soft‐tissue fillers are second in popularity. Common synthetic soft‐tissue filler products include HA fillers and biostimulators, such as calcium hydroxylapatite (CaHA) and PLLA. These agents improve signs of aging by restoring lost volume and stimulating collagen and elastin production [4]. Although HA fillers are generally well‐tolerated, emerging evidence suggests a longer longevity than that initially anticipated. For instance, in a study using magnetic resonance imaging (MRI), HA filler was found to persist in the midface up to 15 years post‐injection. HA was seen on MRI in the deep medial cheek, medial suborbicularis oculi, lateral orbicularis oculi, buccal, pyriform, infraorbital, middle cheek, and temporolateral cheek fat compartments [5]. Master et al. [6] further explored the persistence of HA by performing sequential imaging on a patient who received HA filler for midface volume augmentation, jawline definition, and chin projection. Although chin filler was nearly completely degraded at 19 months, HA in the lateral and midface fat pads remained stable at 27 months after the final injection. These findings suggest that HA filler longevity is influenced by several factors, including injection site, depth, volume, and filler type. Injections of larger volumes into less mobile areas, like the midface, appear to exhibit greater longevity compared to injections of smaller volumes into more dynamic areas, like the chin.

The potential longevity of HA filler has raised concerns among plastic surgeons regarding the impact of repeated filler injections on future surgical interventions, such as facelifts. Concerns include the possibility of prominent product accumulation, local fibrosis, and distorted tissue planes, which could theoretically increase surgical complexity and affect outcomes [3]. Although HA fillers can persist for extended periods, their longevity and impact on facial anatomy are influenced by multiple factors—including injection site, depth, volume, and product type—and strategic placement of small volumes at appropriate intervals is likely to minimize long‐term accumulation and reduce interference with future facial surgery.

Histological evidence additionally supports the overall biocompatibility of HA fillers, which rarely elicit significant inflammatory or granulomatous responses. Although persistent, untreated filler‐related complications could cause fibrotic reactions and tissue distortion, potentially compromising the surgeon's ability to achieve optimal outcomes, such instances are likely rare. This is supported by studies demonstrating a lack of foreign body or fibrotic reactions to subcutaneous or dermal fillers after several years [7, 8]. In 31 cases of incidental filler seen on Mohs micrographic surgery frozen sections, only one case of mild peri‐lymphocytic infiltrate was seen 3 months after being placed to lift an atrophic skin graft [7]. To date, one expert‐guidance publication recommends dissolving HA filler several weeks before facelift surgery if the product lies within the anticipated dissection planes [9]; however, the recommendation is opinion‐based, as no outcome data currently demonstrate that pre‐operative hyaluronidase improves surgical difficulty or post‐operative results.

Biostimulatory fillers, PLLA and CaHA, induce neocollagenesis through a controlled, mild inflammatory response leading to secondary fibroplasia and volume rejuvenation [10, 11, 12]. Some surgeons express concern that, by stimulating fibroblasts, biostimulators may induce tissue remodeling and impede efficient dissection of tissue planes during facelifts. However, available evidence suggests a limited impact. In fact, a large case series of 190 patients found that PLLA injections resulted in a thickened, firmer superficial musculoaponeurotic system (SMAS) that not only did not impede SMAS elevation, but was thought to enhance the durability of SMAS suspension [1]. PLLA has also been demonstrated to help restore the deteriorating skeletal framework with time [13], which may provide further support to overlying soft tissues and contribute to more durable and robust facelift results.

Post‐operatively, PLLA may be a useful adjunct for addressing residual or recurrent volume loss. Although fat grafting is frequently performed during facelift surgery (reported in up to 85% of procedures in a 2015 survey) [14], its variable survival and retention rates (reported at 20%–90%) remain significant limitations [15]. Since fat grafts are biologically active, they undergo expected volume changes that accompany normal aging and weight fluctuations [16]. Early findings suggest a synergistic effect between fat grafting and PLLA in enhancing tissue healing, collagen formation, and facial volumization post‐surgery [1]. In a study exploring patient demand for PLLA post‐facelift.

In one study, 79% of facelift patients later received PLLA for volume restoration, most often 12–18 months after surgery, highlighting ongoing patient demand for nonsurgical maintenance and refinement [1].

A study by O'Daniel et al. [1] explored patient demand for PLLA post‐facelift. In their analysis of 241 patients who received facelift surgery, fat grafting, and PLLA injections at their practice, 79% received PLLA after their surgery. The mean time between facelift with fat transfer and PLLA injections was 14 months. The primary indications for PLLA included unexplained early graft loss, significant post‐operative weight loss, normal aging‐related volume loss, high lean body mass, and the need to address areas not adequately treated via fat transfer. The majority of patients who received facelifts and fat transfer and PLLA at their practice sought PLLA after their facelift, suggesting ongoing demand for volume replacement and refinement after surgery. Subtle volume deficits may become increasingly apparent post‐operatively, potentially related to tissue undermining, particularly in areas like the lateral cheek, where fat pad resorption may occur and preauricular PLLA can offer benefit.

Although rare, surgical facelifts are reported to include a range of potential complications including temporary or permanent facial nerve injury, hematoma, skin necrosis, seroma, or infection [16]. In a study evaluating 11 300 surgical facelift cases, hematoma was the most common complication (1.1% of patients), followed by infection (0.3%), and then by pulmonary or cardiac dysfunction, wound‐related complications, and suspected or confirmed venous thromboembolism in < 0.1% of patients [17]. In our study of 20 patients receiving a surgical facelift at an outside office, one patient reported an infection, with no other aforementioned complications being reported within our patient cohort.

Our study, which included 18 patients with a history of prior HA filler and 11 patients with a history of prior biostimulator filler, did not encounter any surgical complications related to prior filler treatments. Contrary to the myth that filler should be dissolved prior to facelift surgery, none of the patients in our study required filler dissolution prior to their procedure. These observations, alongside limited histologic evidence of significant inflammatory or fibrotic reactions to HA fillers, suggest minimal potential for major surgical complications related to prior filler use. Of note, the only patients who reported scarring following facelift surgery were those who underwent multiple facelift procedures. Previous PLLA treatment is even suggested to contribute to more durable and predictable facelift and fat transfer results as a pre‐treatment and maintenance agent [1].

Notably, 12 out of 20 patients within our study sought additional filler treatment on average 12–14 months after their facelift, with some seeking treatment as early as 31 days post‐operative. No significant difference in post‐surgical filler treatments between patients who did and did not receive fat grafting was seen. Our findings and those of O'Daniel et al. underscore a strong patient demand for volume replacement after receiving facelift surgery.

Our literature search revealed limited data regarding EBDs and facelift surgery. EBDs, such as monopolar radiofrequency (MRF) and MFU, target age‐related tissue laxity. They produce variable amounts of tissue tightening by delivering focused thermal energy to target structures, which induces immediate collagen contraction and denaturation, initiating a process of tissue remodeling and new collagen synthesis [18]. In a recount of a plastic surgeon's experience, MRF was safely performed during facelift procedures as an ancillary treatment to enhance final outcomes [19]. Within our study, 13 patients received MRF or MFU treatments before their facelift, and three patients sought MRF or MFU treatments following their facelift. No surgical complications related to prior EBD treatments occurred in this cohort.

This study has several limitations, including its small sample size, retrospective design, and absence of a control group. Variability in surgeon technique and potential recall bias further limit generalizability. Statistical analyses done were descriptive in nature and not powered to detect causal associations. Additionally, product‐level injectable details (product type, injection volume, injection plane) were inconsistently documented, and little empirical evidence exists to clarify how these variables might affect facelift dissection or post‐operative outcomes. Because injection sites and volumes had not been uniformly recorded, we were unable to perform anatomical mapping or assess whether injection patterns changed following surgery. Future studies should incorporate objective measures of skin and volume changes, standardized photography, surgeon‐reported assessments of dissection difficulty, and standardized injection documentation to better clarify the true clinical impact of prior injectable and EBD use.

## 5 Conclusion

Facial rejuvenation requires an integrated approach to address the complex interplay of physiologic changes contributing to facial aging. Although facelift surgery remains a cornerstone in addressing skin laxity and sagging, it does not fully restore youthful volume or improve skin quality. Fat grafting, although beneficial, has limitations related to graft survival and volume maintenance. The adjunctive utilization of injectables, biostimulators, and EBDs may complement surgical procedures by enhancing contour, promoting collagen remodeling, and maintaining long‐term results.

In this small retrospective cohort, the prior use of injectables or EBDs was not associated with an observed increase in facelift complications. However, given the limited sample size, retrospective design, and heterogenous surgical techniques, these findings should be considered preliminary. The absence of major complications in this cohort cannot confirm safety but rather supports the need for future studies to validate these early findings.

Importantly, understanding how different injectable classes, such as HA, PLLA, and CaHA, in different anatomical locations, such as superficial or deep placement, interact with facial anatomy and surgical planes over time will be critical for establishing best‐practice recommendations. Collaborate efforts between dermatologists and plastic surgeons will be essential to define evidence‐informed strategies to optimize patient outcomes through safe and thoughtful integration of surgical and nonsurgical facial rejuvenation techniques.

## Author Contributions

Authors S.D. and S.F. conceived the study and designed the methodology. Authors P.K. and M.F. collected and analyzed the data and interpreted the results. Author B.M. drafted the initial manuscript and performed the literature search to provide a thorough background and review on this topic. All authors contributed to revisions and approved the final manuscript.

## Funding

The authors have nothing to report.

## Disclosure

All authors have been involved in substantive work leading to the manuscript and hold themselves jointly and individually responsible for its content.

## Consent

Consent for the publication of all patient photographs and medical information was obtained by the authors at the time of article submission to the journal, stating that all patients gave consent for their photographs and medical information to be published in print and online with the understanding that this information may be publicly available.

## Conflicts of Interest

The authors declare no conflicts of interest.

## Supporting information


**Appendix S1:** Survey questions.

## Data Availability

The data that supports the findings of this study are available in the Supporting Information of this article.
