# Supplementary figures and images for: Injectables and Facelifts: Can We Coexist? A Retrospective Chart Review Assessing Injectable Treatments Preceding and Following Rhytidectomy
Source: J Cosmet Dermatol. 2026 Feb 9;25(2):e70690. doi: 10.1111/jocd.70690 (PMC12887548; doi:10.1111/jocd.70690)

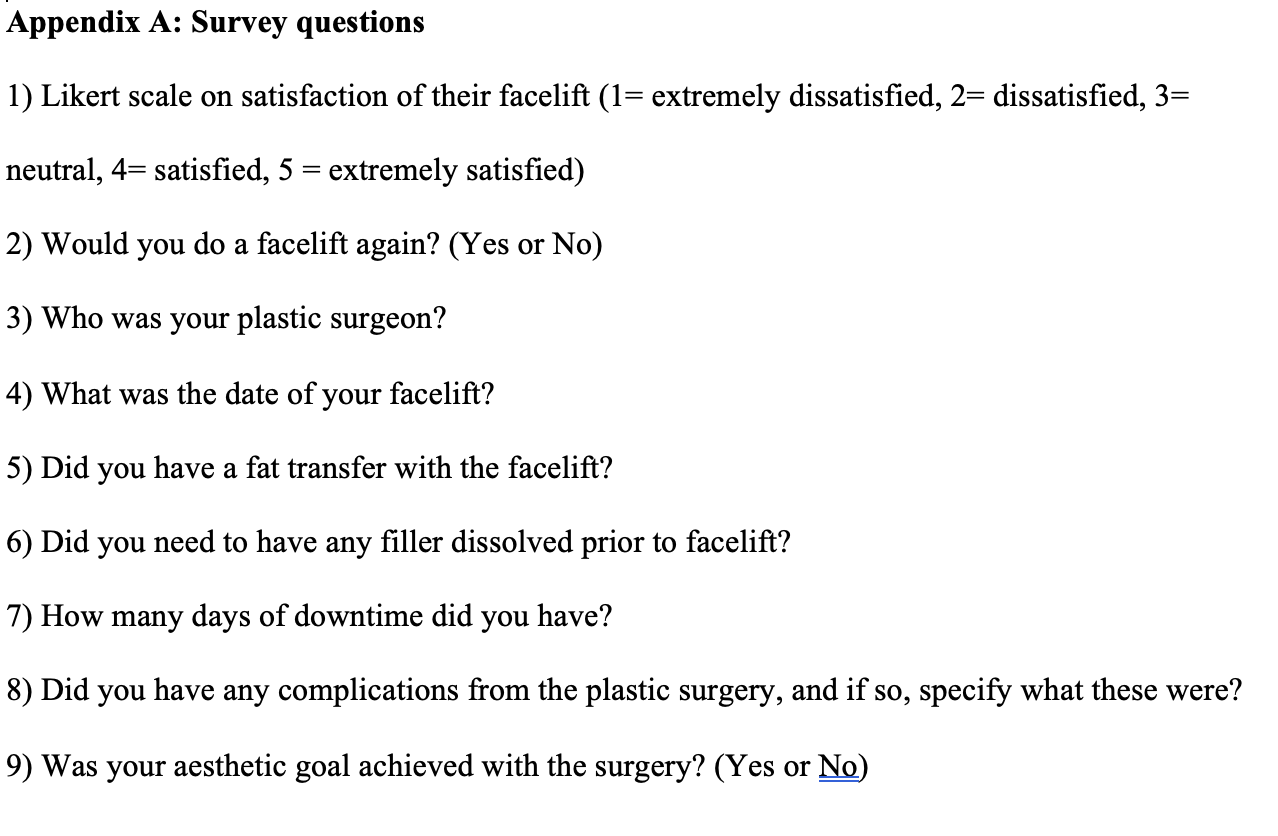

Supplement: Supplementary file 1 — Appendix S1: Survey questions. [file JOCD-25-e70690-s001.png]
